# Utilization of dielectric properties for assessment of liver ischemia-reperfusion injury in vivo and during machine perfusion

**DOI:** 10.1038/s41598-022-14817-3

**Published:** 2022-07-01

**Authors:** Jie Hou, Olav Magnus Ivar Liavåg, Ida Høy Færden, Ørjan Grøttem Martinsen, Tor Inge Tønnessen, Pål-Dag Line, Morten Hagness, Jan Olav Høgetveit, Søren Erik Pischke, Runar Strand-Amundsen

**Affiliations:** 1 Department of Physics, University of Oslo, Sem Sælands vei 24, 0316 Oslo, Norway; 2 Department of Clinical and Biomedical Engineering, Oslo University Hospital, 0424 Oslo, Norway; 3 Section for Transplantation Surgery, Department of Transplantation Medicine, Oslo University Hospital, 0424 Oslo, Norway; 4 Department of Emergencies and Critical Care, Oslo University Hospital, 0424 Oslo, Norway; 5 Institute of Clinical Medicine, University of Oslo, 0318 Oslo, Norway; 6 Department of Immunology, University of Oslo, 0372 Oslo, Norway; 7 Division of Technology and Innovation, Oslo University Hospital, 0424 Oslo, Norway

**Keywords:** Diagnosis, Hypoxia, Biological physics, Tissues, Gastroenterology, Medical research

## Abstract

There is a shortage of donor livers and patients consequently die on waiting lists worldwide. Livers are discarded if they are clinically judged to have a high risk of non-function following transplantation. With the aim of extending the pool of available donor livers, we assessed the condition of porcine livers by monitoring the microwave dielectric properties. A total of 21 livers were divided into three groups: control with no injury (CON), biliary injury by hepatic artery occlusion (AHEP), and overall hepatic injury by static cold storage (SCS). All were monitored for four hours in vivo, followed by ex vivo plurithermic machine perfusion (PMP). Permittivity data was modeled with a two-pole Cole–Cole equation, and dielectric properties from one-hour intervals were analyzed during in vivo and normothermic machine perfusion (NMP). A clear increasing trend in the conductivity was observed in vivo in the AHEP livers compared to the control livers. After four hours of NMP, separations in the conductivity were observed between the three groups. Our results indicate that dielectric relaxation spectroscopy (DRS) can be used to detect and differentiate liver injuries, opening for a standardized and reliable point of evaluation for livers prior to transplantation.

## Introduction

The waiting lists for liver transplantation are increasing worldwide, accompanied by climbing waiting list mortality^1,2^. To change this negative trend and to expand the number of transplantable livers, the utilization of marginal donors with potentially inferior liver quality has increased^3,4^. The margins for what constitutes a “transplantable liver” are presently subject to research, where conditioning and evaluation of liver parameters are important inputs for decision-making. Human donor livers, especially those procured from marginal donors, can have various degrees of injuries that may either come from before the procurement (e.g fatty liver, cirrhosis) or during the procurement (warm ischemia injury). Moreover, donor livers will also experience a certain degree of injury after the procurement during the time of organ preservation outside the body^5^. This may lead to profound ischemia-reperfusion injury at the time of transplantation and consecutively to undesired outcomes like ischemic cholangiopathy, primary nonfunction (PNF) and death of the recipient^1,6^. Transplanting organs under these high-risk circumstances require development of new and more precise tools to assess the transplantability of donated organs, and to potentially mitigate the negative effects of the procurement process and of the organ preservation outside the body^7–10^.

Currently, the common method of evaluation of a liver graft for transplantation, is based on visual inspection of the donor organ by the transplant surgeon, combined with knowledge about the donor history and serology taken before procurement. When in doubt a histological evaluation is carried out, but this technique is time consuming and outcome uncertain. When the surgeon is in a quandary about the condition of the organ, which is a recurrent challenge when assessing marginal liver grafts, these organs will typically be discarded^6^. Approaches using scores (VITTAL and Groningen) with lactate clearance, bile production and perfusate pH stabilization is a matter of ongoing research^11^. The precision of these scores in assessing liver viability and quality is yet to be established^11^ due to the multi-factorial considerations for liver transplantability^6^.

Over the past 20 years, ex vivo machine perfusion of liver grafts has emerged as a platform for organ preservation that hopefully will alleviate all the above-mentioned obstacles in liver transplantation. Contemporary machine perfusion practice in the clinical setting of organ transplantation often comprises a sequential perfusion of the liver graft with cold fluid at temperatures between 4–12 $^{\circ }$C and oxygen (hypothermic machine perfusion (HMP)) for reconditioning of the liver tissue. Thereafter, followed by warm perfusion at 37 $^{\circ }$C (normothermic machine perfusion (NMP)) with blood or blood-like perfusate to reactivate the organ metabolically. The liver is then evaluated to assess the level of graft function and quality^10,11^. This technique has demonstrated superior outcome compared to the gold standard of ex vivo organ preservation called static cold storage (SCS) where the organs are preserved ex vivo submerged in static and cooled (4 $^{\circ }$C) fluid. However, validated guidelines for liver graft function and viability assessment during ex vivo machine perfusion are scarce^6,11,12^. Presently there is ongoing research to investigate if machine perfusion can be used to improve the state of marginal livers and provide accurate information about the condition of the organs^13,14^.

Numerous studies investigating the electrical properties of organs and tissues have been conducted to assess organ condition and to differentiate normal from pathological tissue^15–18^. However, none of those have investigated different liver conditions both in vivo and on machine perfusion using dielectric properties in the GHz frequency range. While low frequency measurements (0.1 Hz–10 MHz) can be sensitive to structural changes in the tissue, high frequency measurements in the GHz region are sensitive to concentrations and distributions of polar molecules. Most of the studies of liver tissue have used instruments in the lower frequency ranges, while only a handful of the studies have used dielectric relaxation spectroscopy (DRS) in the higher frequency ranges^19^. In 2007, O’Rourke et al.^20^ investigated the dielectric properties of human liver, comparing normal tissue, malignant cancer tissue and cirrhotic tissue. Measurements were performed both in vivo and ex vivo from 500 MHz to 20 GHz. They reported that there are no statistically significant differences between the dielectric properties of malignant liver cancer tissue and normal liver tissue in vivo. Furthermore, they stated that in vivo data cannot be represented in terms of a one-pole Cole–Cole model and that further work was needed to uncover the underlying mechanisms in the in vivo liver permittivity data. Farrugia et al.^21^ reported characteristics of the dielectric properties of rat liver from 500 MHz to 40 GHz in vivo and used one Cole–Cole equation to model the measurement data. They suggested that the difference between in vivo and ex vivo (few minutes after excision) dielectric properties can be attributed to tissue hydration. Moreover, Peyman et al.^22^ investigated variations in dielectric properties from 100 MHz to 5 GHz due to pathological changes in the human liver at 25 $^{\circ }$C ex vivo, and they found that cirrhotic livers and liver tumors have higher permittivity and conductivity values compared to normal liver, whereas steatosis resulted in lower permittivity and conductivity. Most recently, Hou et al.^23^ investigated the dielectric properties of pig small intestinal segments with different degrees of ischemia-reperfusion injuries. They reported that the DRS technique appears to be a promising method for assessing the perfusion/reperfusion state of the small intestine, and that machine learning methods can be used to more accurately differentiate viable and non-viable intestinal segments. Currently, there is still a lack of dielectric data at high frequencies from biological tissues, especially on organs undergoing ischemia-reperfusion injuries^19,24^. The present work is an exploratory study, aimed to demonstrate a proof of concept that the DRS technique has the potential to be used in the assessment of liver conditions, both in vivo and during machine perfusion.

Porcine livers were used in this study, as they are close to the human liver with respect to functionality and anatomy^25^. We developed and characterized a dielectric profile over the frequency range 200 MHz–14 GHz over a four-hour duration, for both healthy liver and biliary injured livers in vivo. In addition, we characterized the dielectric properties during four hours of NMP on control (CON)-, biliary injured (AHEP)-, and overall hepatic injured (SCS) livers. In this study, our aim is to investigate the feasibility of measuring DRS on livers both in vivo and during machine perfusion, to study the association between dielectric properties on livers with different levels of injury.

## Results

**Figure 1:**
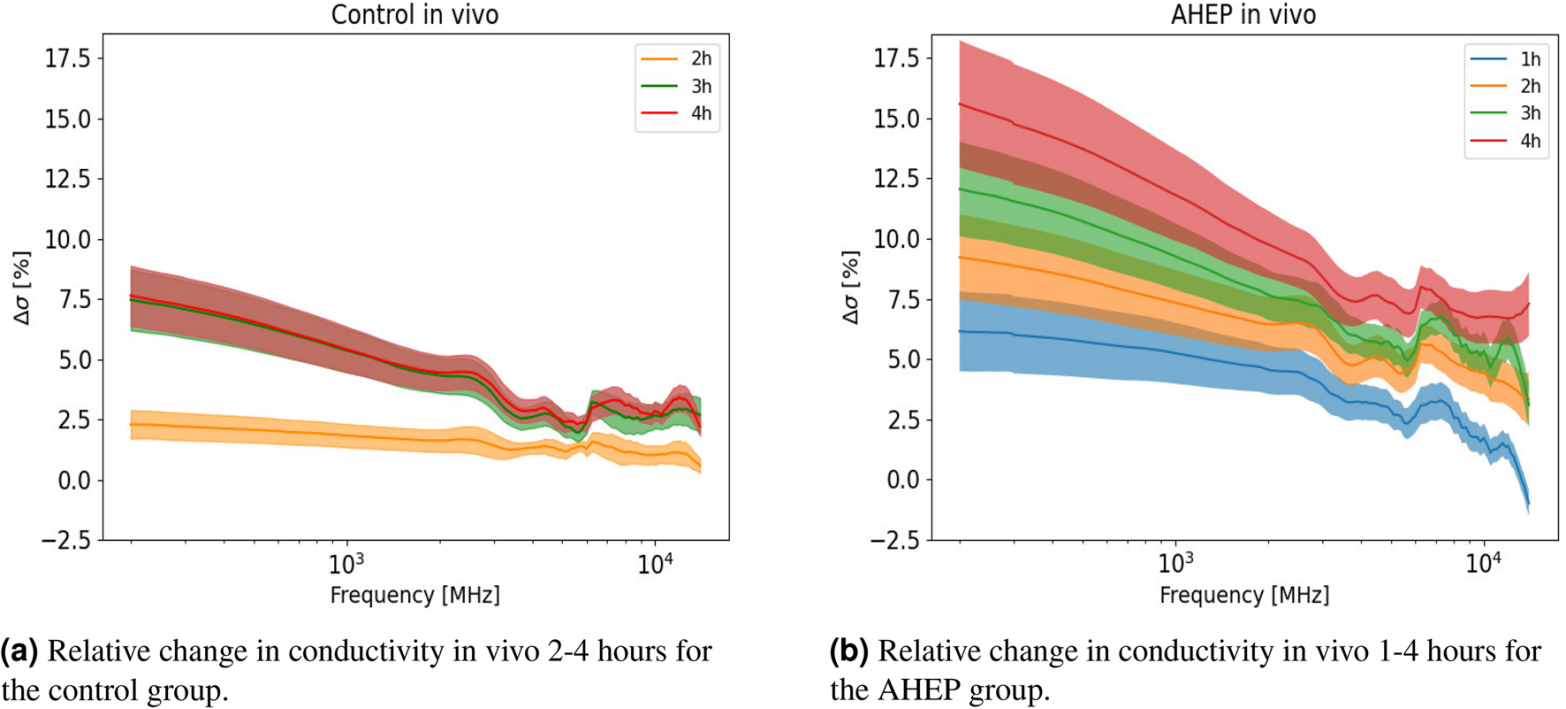
Figure shows the relative change (mean and standard error) in conductivity monitored in vivo for both control and AHEP livers calculated with Eq. (2). One hour data is not shown in (**a**) as it is used as reference data (baseline at value zero on the y-axis). Frequency range used was 200 MHz–14 GHz (plotted in a logarithmic scale on the x-axis). N = 7 for each of the groups.

**Figure 2:**
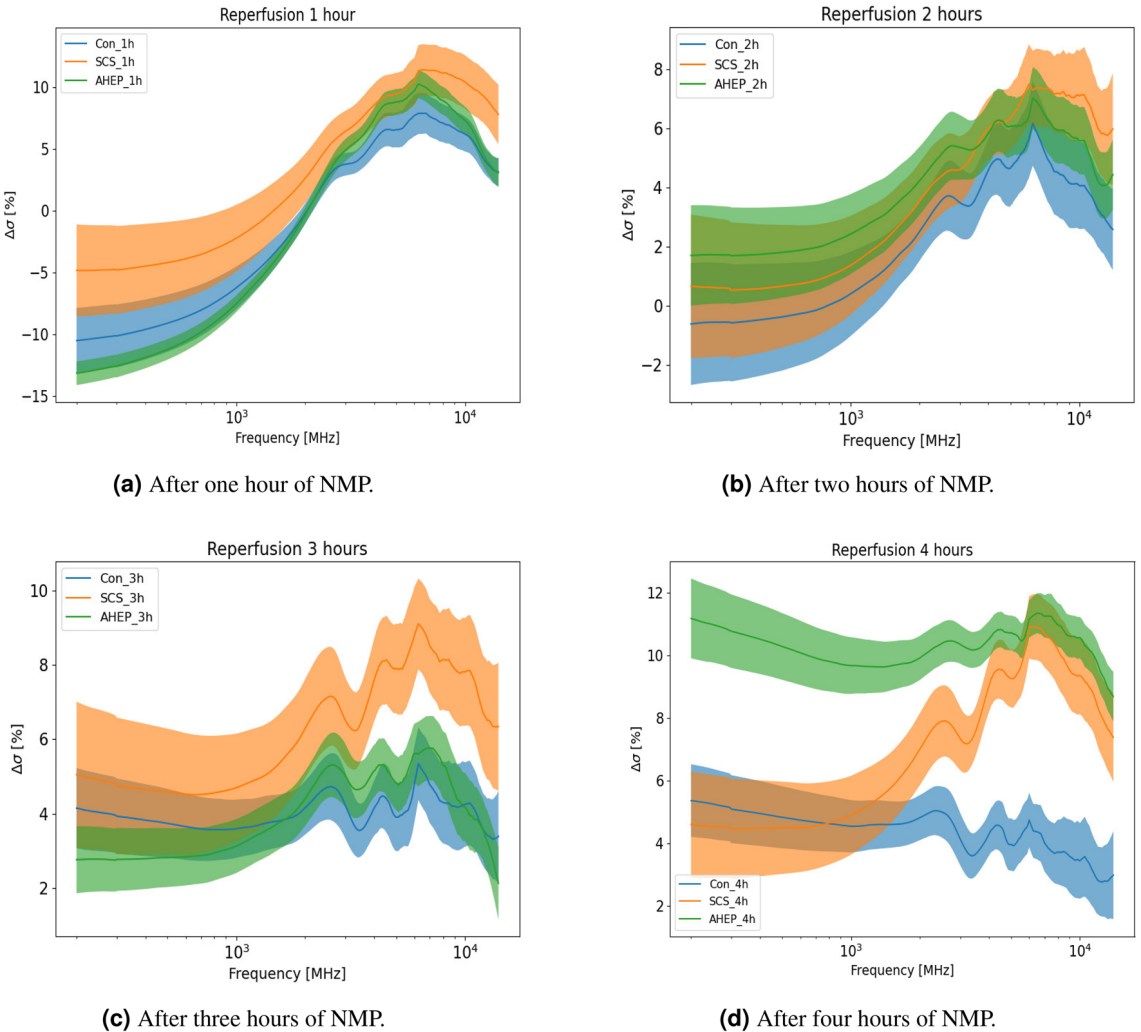
Relative change in the conductivity for three groups as function of frequency displayed with mean and standard error calculated with equation  (2). Frequency range used was 200 MHz–14 GHz (plotted in a logarithmic scale on the x-axis). N = 7 for each of the groups.

During the in vivo measurements, although not being a predefined end-point or subjected to systemically evaluation in this study, we did not observe any visual changes of the liver surface between the control and AHEP groups. To increase readability, Figs. 1 and 2 show the relative change in conductivity (related to dielectric loss). Plots of raw conductivity data can be found in the supplementary material. During the in vivo phase, we observed that there was an initial increase in the relative conductivity of 2.5% after two hours and a further increase of 7.5% after three and four hours for the livers in the control group (Fig. 1a) in the lower frequency region. The AHEP group showed a significant larger increase in the conductivity in vivo compared to the control livers where no intervention was performed. The conductivity increased in the AHEP group from around 5% to almost 17% in the lower frequency region. In the higher frequency region, the conductivity increased approximately 8% from the starting level during the four-hour in vivo phase (Fig. 1b).

For the in vivo data, we performed hourly comparisons (four hours) within each of the groups (control and AHEP). Significant differences were found ($p < 0.05$) in all paired comparisons for one to four hours of the in vivo phase for the control group, except for data from one hour compared to data from two hours (Table 1). For the AHEP group, statistical significant differences were found in all hourly paired comparisons, except for two h﻿ours compared to three h﻿ours (Table 1). The control group was significantly different compared to the AHEP group at all time points, except at the one h﻿our time-point (Table 2).

**Table 1:** Hourly based pairwise comparison for livers in the control and the AHEP group in vivo. The table shows the frequency ranges where statistical significance were found in each hourly pairwise comparison, for livers in the control group and AHEP group (comparison within the same group). “*NS*” = “Not Significant”, “*N/A*” = “Not Applicable”.

Control	1 hour	2 hours	3 hours
**2 hours**	NS	N/A	10.75–14 GHz
**3 hours**	9.50–14 GHz	10.75–14 GHz	N/A
**4 hours**	8.25–14 GHz	10.0–14 GHz	NS

AHEP	1 hour	2 hours	3 hours
**2 hours**	12.25–14 GHz	N/A	NS
**3 hours**	10.0–14 GHz	NS	N/A
**4 hours**	8.50–14 GHz	12.0–14 GHz	13.25–14 GHz

**Table 2:**
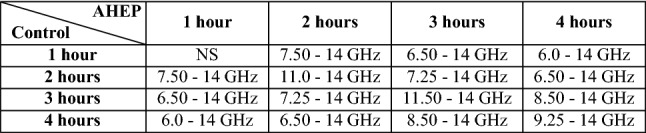
Pairwise comparison for in vivo data at one-h﻿our time interval. Table show the frequency ranges where statistical significance were found in each hourly pairwise comparison, between livers in the control group and the AHEP group (Comparison between two different groups). “*NS*” = “Not Significant”, “*N/A*” = “Not Applicable”.

After organ procurement, cold dual hypothermic oxygenated perfusion (DHOPE) and controlled oxygenated rewarming (COR) were performed, followed by the NMP procedures. Dielectric conductivity was measured for the three groups after one, two, three and four h﻿ours of NMP and compared to the in vivo control group (Fig. 2). Following one hour of NMP, the changes in conductivity appear similar in frequency dependence for the three groups, with some variations in amplitude. Compared to the in vivo control reference, the conductivity decreased around 5–14% in the low frequency region, while increasing about 5–13% in the high frequency region (Fig. 2a). Following two h﻿ours of NMP, the overlap between the three groups increased in the conductivity spectrum over the whole frequency range, while the curve shapes remained similar to the results after one h﻿our (Fig. 2b). Following three hours of NMP, the SCS group differed from the other two groups, with higher conductivity in the high frequency region (1–14 GHz) (Fig. 2c). There was no statistical significant difference between the three groups following one, two and three h﻿ours of NMP. After four hours, there was a clear separation in the conductivity spectra between the three groups.

The differences between the NMP control group and the in vivo control reference appeared to be decreasing after four h﻿ours of NMP. The conductivity of the AHEP livers was significantly higher than the control livers following four hours of NMP (Fig. 2d). The SCS livers were similar to the control livers at low frequencies, and similar to the AHEP livers at the highest frequencies (7–14 GHz) (Fig. 2d). There were statistically significant differences between the control group and the AHEP group in the frequency ranges 4.75–5.3 GHz and 6.25–12.5 GHz, and between the control group and the SCS group in the frequency range 6.25–8.25 GHz after four h﻿ours of NMP (Fig. 2d).

**Figure 3:**
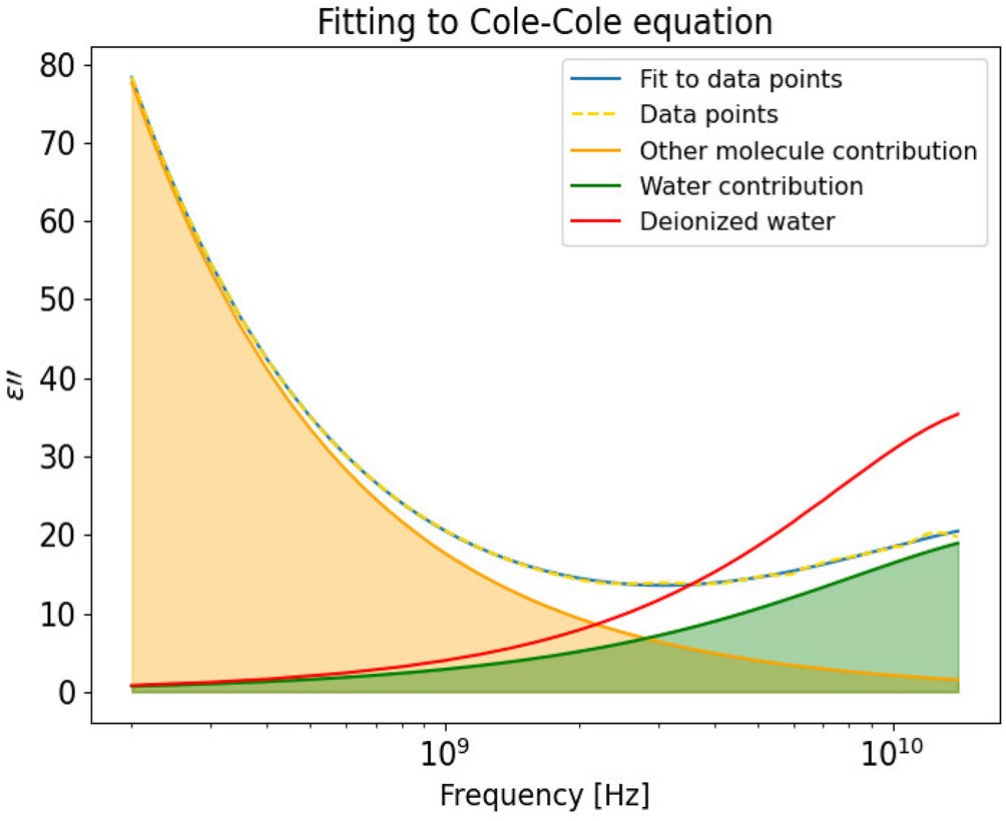
Cole–Cole model fit to data from 200 MHz to 14 GHz including 167 data points and separation of the two processes together with dielectric loss of deionized water.

An example of fitting the control liver in vivo data to a two-pole Cole–Cole equation (Eq. (3)) is shown in Fig. 3. The orange curve is the contribution from the larger molecules (compared to water molecules) and the green curve is the contribution from the water molecules, whereas the red curve indicates the dielectric loss for pure deionized water measured at 37.5 $^{\circ }$C. The fitting results are similar for the other groups and are not shown.

**Figure 4:**
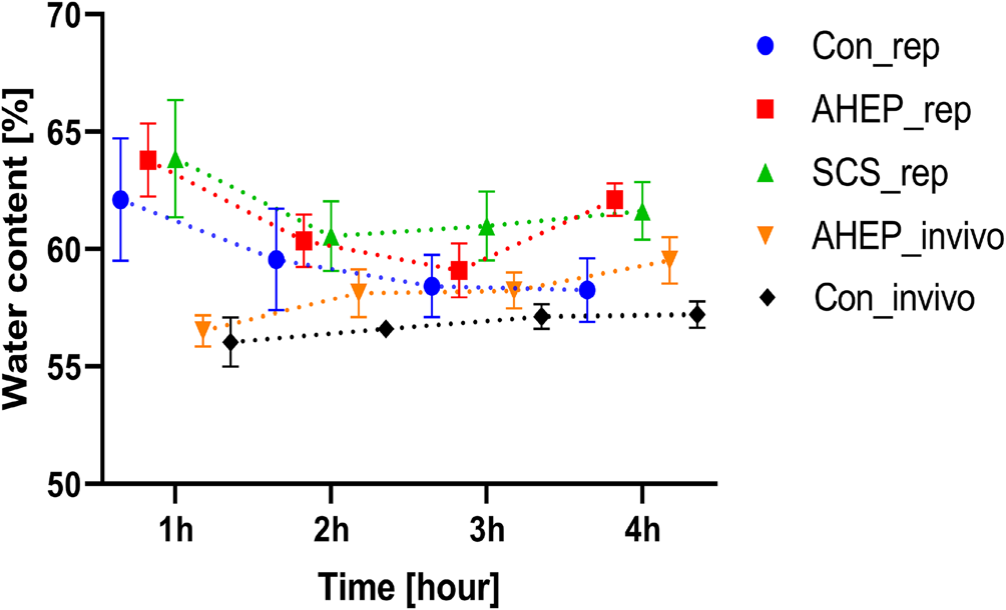
Comparison of calculated mean water content with standard error among the groups: Control, AHEP and SCS during both the NMP and the in vivo phase. “rep” = “reperfusion phase during NMP”, “invivo” =“in vivo phase”.

The calculated water content was compared between the three groups with NMP and in vivo measurements from the control and the AHEP groups (Fig. 4). The water content was calculated based on the Cole–Cole parameters (A complete table of extracted Cole–Cole parameter values with 95% confidence interval (CI) is provided in the supplementary material). All $\alpha$ values obtained were lower than 0.2, all $\alpha _1$ values were around 0.06 and most of the $\alpha _2$ values were around 0.16. The coefficient of determination $R^2$ for the Cole–Cole parameters were all above 0.9998.

During the in vivo phase, the water content remained nearly constant for livers in the control group while an increasing trend was observed for livers in the AHEP group. During NMP, the water content was highest after the first hour. Following two hours of NMP, all three groups displayed a decreasing trend. After three and four hours of NMP, the water content in the control group continued to decrease, while the water content in the SCS group remained high. The water content in the AHEP livers decreased during the first hours of NMP, similar to the control group, but then started to increased towards the end of the NMP (Fig. 4).

We measured the weight of the livers two times during the experiment, once immediately after explantation and once after machine perfusion. For livers in the control group, the average weight was 1666.9 g before machine perfusion and 1755.3 g after machine perfusion, with an average increase of weight 88.4 g. For livers in the AHEP group, the average weight was 1648.9 g before machine perfusion and 1774.3 g after machine perfusion, with an average increase of weight 125.4 g. For livers in the SCS group, the average weight was 1625.7 g before machine perfusion and 1790.0 g after machine perfusion, with an average increase of weight 164.3 g. The weight increase after machine perfusion was highest for livers in the SCS group, followed by the AHEP group and the lowest increase in weight was the livers in the control group. This result is consistent with the water content estimation showed in Fig. 4 after four hours of NMP.

## Discussions

Dielectric measurements were performed in vivo and during NMP on livers from 21 pigs. We compared the dielectric properties from three groups of livers undergoing NMP (healthy liver with no injury, liver with biliary ischemic injury and liver with global hepatic ischemic injury), with the dielectric properties of the in vivo control liver data as reference. Significant differences in the time development of dielectric conductivity were found in vivo in AHEP livers, and after four hours of NMP between all three groups. Analysis of the dielectric data enabled differentiation between injury groups during NMP.

Occlusion of the hepatic artery for four hours leads to gradual injury of the bile ducts, while the parenchyma is preserved^26,27^. Bile duct injury occurs because the biliary tree is supplied mainly by the hepatic artery, whereas the liver parenchyma is supplied by both hepatic artery and through the partially oxygenated blood of the portal vein. Static cold storage causes diffuse liver injury over time because it is a cold ischemic condition. The increased conductivity observed in these livers (AHEP) compared to the control group (Fig. 1b) may be related to an ischemia induced rise in the concentration of larger molecules, like lactate^28^ (which is a dipolar molecule). When the concentration of dipolar molecules increases, there will be a higher level of polarization in the tissue, as there are more polarizable molecules present. The dipolar molecules will rotate to follow the alternating electric field, where the rotational movement causes increased energy loss which is directly proportional to the dielectric conductivity. Therefore, we speculate that the increase in the conductivity shown in Fig. 1b is due to an increase in concentration of dipolar molecules.

During the machine perfusion, the largest changes compared to the in vivo control group, occurred after one hour of NMP (Fig. 2a). This was expected as the liver tissue is in an altered condition after static ischemia, cold perfusion and slow rewarming, most possibly leading to an initial reaction that strongly influences the dielectric properties of the liver. After the initial changes that were similar in all three groups, the differences in dielectric properties between the groups increased during the fourth hour of normothermic perfusion. This might be related to the gradually appearing consequences of ischemia-reperfusion injury and the advancing metabolic activity, which was returning in control livers, but was hampered in the intervention groups. As known from other studies on ischemia-reperfusion injury, this is not a binary event, but reperfusion injury is gradually developed based on several processes that occur following the return of oxygenation and sufficient blood flow^29–34^. Following three hours of NMP, the dielectric changes in the SCS group stands out compared to the other groups, which may be related to a higher level of ischemic injury in the SCS group. After four hours of NMP, the dielectric conductivity values of the NMP control group appears to approach the values of the in vivo control group (Fig. 2d).

Our results visualize significant measurement frequency and time dependent differences during NMP between all three groups (Fig. 2d). Based on these results we suggest that the phase with NMP plays an important role in order to be able to evaluate the liver condition, and that four hours of NMP is needed to be able to differentiate between different ischemia-reperfusion liver injuries using DRS. In the upcoming study (on human livers that have been discarded for transplantation), we will investigate if this technique can be applied with shorter machine perfusion time and also study the trends with longer perfusion time (eight hours).

During in vivo normal perfusion, water content is tightly regulated across the tissue, and a balance is upheld between intracellular, extracellular and vascular liquids. Exposure to ischemia and reperfusion injuries influence this balance and affects how liquid is distributed in the tissue^29,30^. This can be in the form of changing the balance between intra- and extra-cellular liquids, as well as congestion of extracellular liquids or formation of edema. Thus, water content and distribution are related to tissue state.

In this study, we propose a method for non-destructive water content quantification, where we use the dielectric properties of deionized water to indirectly calculate the water content in the liver. The reported water content of normal pig liver is 74.5 ± 1.3%^35^, 71–75%^36^, 64.17%^37^ and 69 ± 1.5%^38^. Our DRS based method of water content quantification estimated an average value of 56.05% in the control livers. Underestimation of the liver water content using the DRS method could be due to the fact that water molecules in tissue are influenced/bound by forces from dissolved materials and fixed structures that are not present in deionized water (free and isolated water molecules).

The water molecules in tissue can be polarized by nearby biomolecules and ions, forming sheets around these molecules. Polarized water molecules can again cause polarization in their nearby neighboring molecules. The effect of the polarization on the water molecule is called “slow orientational dynamics”^39^. The slowing down of the orientational dynamics of water molecules associated with ions and biomolecules is mainly due to solvation mechanics (hydrogen bonding, electrostatics, and confinement effects)^39^. Thus, the time constant of the movement of polarized water molecules will be different than that of free water molecules. This influences the frequency dependent behavior of the measured dielectric properties, which may have led to an underestimation of the calculated water content. Most of the water inside living cells have picosecond orientational dynamics^39^. This is consistent with our calculated water relaxation time $\tau _2$ which ranged from 5 to 9 picoseconds.

While acknowledging that our method underestimates the absolute water content, still a comparison of the relative changes of water in the different groups is of interest. We assume that the underestimation affects each group equally and that this method of estimation reveals the changes in water content in the liver tissue over time. The first decreasing trend in the estimated water content parameter for the three groups ex vivo appears from the beginning of NMP, where we believe the initial high water content could be related to the forgoing SCS and cold perfusion. For livers in the AHEP and SCS groups, the decreasing trend was followed by an increasing trend after three and two hours of NMP, respectively. This may be related to ongoing ischemia-reperfusion injury which leads to edema creation^29^. In comparison, the control livers showed a consistent decreasing trend during NMP towards the in vivo levels of the control livers. Likewise, in vivo hepatic artery occlusion leads to a consistent increasing trend of liver water content compared to the control livers and might be the result of the partial ischemia induced by the hepatic artery occlusion. The association between DRS based estimation of liver water content and the measured weight differences between pre- and post-NMP livers, shows that this exploratory method (DRS) for estimating tissue water content can potentially be used to assess the edematous condition of the liver. More experiments with longer machine perfusion time is needed to determine whether use of the estimated water content parameter can have significant clinical impact.

DRS is typically used to characterize solid materials or liquids, and have over the years also been used to characterize biological tissue. Despite the advantages of DRS with the OCP technique, the main challenge associated with this method is the repeatability of the data. One major issue is that there is a large variability in the published dielectric data between different studies and between different measurements on the same tissue^19,40^, possibly due to different instrumentation and measurement techniques used. Among the more apparent causes for this variation is the observation that these measurements have been performed under varying conditions, both on ex vivo and in vivo tissue, and at different points in time with respect to tissue perfusion and temperature.

Most DRS studies have been carried out ex vivo, yet the dielectric properties of ex vivo tissue do not necessarily reflect properties of in vivo tissue. Thus, there is a need for comparing dielectric properties of both in vivo and ex vivo tissue from the same subject^24^. This study assessed dielectric parameters of the liver, measured over a wide frequency range, both in vivo and ex vivo, on livers with varying levels of ischemia and reperfusion injury. It may thus serve as reference for future studies aiming at diagnosing liver injury. Knowledge gained from this study enables us to choose optimal measurement frequency regions for future studies. Especially the high frequency region is of interest as we found statistical significant differences between the intervention groups in this region. Results from this study also indicate that three to four hours of NMP is needed to reliably assess differences in dielectric liver parameters that are associated with pre-existing differences in injury levels and tissue state. In addition, our results can be used to differentiate between liver injury types during NMP and can be a relevant method for enabling an objective evaluation of livers prior transplantation.

In this study, we used the DHOPE-COR-NMP protocol^41^ of machine perfusion with the aim to assess livers after an optimal conditioning and reperfusion of the liver graft. The Groningen group established this protocol to allow both restoration of mitochondrial activity during hypothermic preservation, minimize reperfusion injury by gradually rewarming and assessing function during normothermic perfusion, which has been described in clinical trials^11,42^.

The intervention groups in this study were established with the aim to evaluate distinct model liver injuries. The intention of using the AHEP model is to create biliary injury, while leaving the liver parenchyma relatively unharmed, as it receives partly oxygenated blood through the portal vein. Due to biliary complications and late onset, non-anastomotic strictures is a major concern in a high proportion of marginal liver grafts, in particular grafts subjected to prolonged warm ischemia, it would be beneficial to have a model to address this complication. Furthermore, AHEP is of interest as it is relevant for the most serious complication following liver transplantation, where thrombosis occurs in the hepatic artery. This model may be comparable to hepatic artery thrombosis which is a well-established cause for bile duct injury in liver transplantation^43^. Thus, we utilized a model with a reproducible extent of biliary ischemic injury through the AHEP model. As for the SCS group, prolonged SCS preservation leads to liver graft injury. We aimed to use this approach to model a standardized, generally injured liver, which is difficult to obtain by other methods.

For livers in the control and AHEP group, a range (1–2 h) of SCS was conducted following explantation. The reason for the small variation of the SCS duration for the livers in both Control and AHEP groups was the different time needed by the surgeons and staff to properly cannulate the liver to the perfusion machine.

Regarding the measurements techniques, the probe was placed on the surface of the liver lobe edge, with a measurement depth sensitivity of a few millimeters, while changes that occur deeper in the tissue could not be detected. The rate and degree of discoloring of the liver surface during normothermic perfusion differed between and within liver lobes. This can be attributed to the pig liver anatomy with its thin and separate lobes rendering it especially vulnerable to inappropriate physical positioning ex vivo, leading to heterogeneous and reduced perfusion of different parts of the liver parenchyma^21,44^. This may have influenced our measured data, as we might not have captured all the variations across the liver, and could also not measure the changes that occurred in the deeper layers of the liver. Regarding the sterility, we believe that this technique has a great potential to be applied in a clinical setting, we are currently testing different sterilization methods, for instance using hydrogen peroxide on the probe itself and a sterile camera drape around most of the probe and the communication wire.

For all three groups, the same temperature was used and maintained. Still, there might have been a slight difference in the surface temperature between in vivo and ex vivo measurements. Small changes in the temperature alone do not explain the observed difference in the dielectric properties for the three groups. In addition, there might be some minor natural differences in the composition of the autologous blood used as perfusate during the machine perfusion. The positioning of the liver graft in the organ chamber of the perfusion machine is also of considerable importance in order not to compromise the circulation of the liver parenchyma, especially due to compression or blockage of the liver vein outflows. The size of the liver could furthermore influence its perfusion, for instance in case of bigger liver grafts leading to self compression of liver parenchyma and/or blood vessels due to heavier weight. The livers used in this study were all very similar and well fitting in size regarding the reservoir space, and the positioning of the liver was standardized with the liver laying on its ventral surface in the machine reservoir, with the liver veins pointing towards the back of the perfusion machine. We acknowledge that there might be small variations in the liver size and physical positioning. Every effort was given to make these as similar as possible, and given that the same protocol was used for all livers during machine perfusion, the above mentioned variations cannot explain the differences in the dielectric properties we observed in this study.

Our measurements were performed on porcine models. Peyman et al.^22^ concluded that variation between dielectric properties of liver tissue from human and other species are not greater than variations due to inhomogeneity in each tissue at a range of temperatures. It is challenging to interpret the relationship between the changes in measured conductivity and the specific physiological changes in the liver, since both warm and cold ischemia impose somewhat unpredictable injury on the liver^45^. Categorizing liver ischemia-reperfusion injury is not as straightforward as cirrhosis and steatosis, which present significant visible changes on the liver tissue. Nevertheless, the observed differences between the three groups indicate that DRS has the potential to detect physiological changes in the liver that are not visible to the human eye.

The evaluation of marginal liver grafts is reliant on a comprehensive assessment of liver function. The biliary tree represents the “Achilles heel” of liver transplantation, and although biliary excretory function may be intact, ischemic injury to the biliary tree may manifest late, months after the transplantation. This study is first and foremost a pilot study of how dielectric conductivity assessed by dielectric relaxation spectroscopy is altered in an experimental setting of prolonged static cold storage and biliary ischemic injury in an animal model. Due to the exploratory nature of the study, one of the limitations is that we are not yet able to predict possible clinical benefits compared to the “standard viability assessment” as published in the literature.

This present study serves as a proof of concept, where our goal is to investigate if there are differences in the dielectric properties of livers that can be associated with the injury level of the livers. To the best of our knowledge, this technique has not previously been used on livers in vivo and during machine perfusion, related to liver transplantation. Based on our results, we estimate that this technique has a potential for use in the assessment of ischemia-reperfusion injury in livers. In the next study, we plan to use the DRS technique on human livers that have been discarded for transplantation, where the measurements will be performed in a sterile fashion. We will also use longer perfusion time (eight hours) to study how the dielectric properties change beyond four hours of NMP. As we collect more data in the future, a machine learning model will be built to automatically process the measured data and give predictions on the liver injury condition. Moreover, we will also compare the present results with measurements performed on human livers and other methods of liver state assessment, including metabolomics, proteomics, emerging machine perfusion quality scores and histology.

## Conclusion

This explorative study is the first to investigate and compare the use of the DRS technique on livers in vivo and during machine perfusion. Various degrees of ischemia-reperfusion injury in porcine livers associate with differences in the dielectric properties both in vivo and on NMP. Significant differences in the dielectric properties between healthy control livers, livers with biliary injury induced by hepatic artery occlusion and livers with global hepatic injury were observed after four hours of NMP, indicating that DRS has the potential to be used to assess different levels of liver ischemia-reperfusion injury. We observed a clear increasing trend in the conductivity spectra in the AHEP group during the four-hour in vivo phase while no visual changes could be observed on the liver tissue. The proposed water content estimation approach could be used as a non-invasive method to evaluate levels of liver edema. As this study serves as a proof of concept using a pig model, future studies need to be conducted to further investigate the clinical applicability of this method during liver transplantation, and the translational possibilities on human livers.

## Methods

### Animals and experimental design

This study was conducted using 21 Norwegian Landrace pigs (Sus scrofa domesticus), weight range 54–66 kg. Food was withheld 12 hours prior to surgery but the animals had free access to water. The pigs were pre-medicated with ketamine (40 mg/kg), atropine (0.05 mg/kg), and droperidol (0.65 mg/kg) intramuscularly at the animal housing facilities before transporting to the operating room. Pigs were ventilated and sedated with sevoflurane (1–1.5%) and pain relief was achieved with continuous infusion of morphine (0.5–1 mg/kg/h). No muscle relaxants were used and the pigs were continuously assessed for reaction to nose and hoof-pinching pain. A median laparotomy was performed, the liver hilus identified. If applicable, 5000 units of heparin was injected prior to the hepatic artery occlusion. At the end of the four-hour in vivo phase, 32,500 units of heparin were administered, 2–2.5 liters of blood were drained to be used for machine perfusion, and the liver excised followed by flushing of the liver with two liters of IGL-1 (Institut Georges Lopez-1) and consecutively cold storing of the liver graft in the same fluid. After liver explantation, 500 mg of pentobarbital sodium and 40 mmol of potassium chloride were injected intravenously for euthanasia. The study was approved by the Norwegian Food Safety Authority (FOTS 24454) and conducted in accordance with national animal welfare guidelines. All methods are reported in accordance with ARRIVE guidelines (https://arriveguidelines.org).

Three groups were established:

- Control (CON) (N = 7): Four h﻿ours in vivo monitoring followed by a short period (one–two hours) of static cold storage before machine perfusion after procurement.
- Biliary injury (AHEP) (N = 7): Four hours of partial warm ischemia with hepatic artery occlusion (where the hepatic artery was fully occluded, while the portal venous flow was intact) prior to liver procurement followed by a short period (one–two hours) of static cold storage before machine perfusion.
- Global hepatic injury (SCS) (N = 7): Four h﻿ours in vivo monitoring followed by static cold storage for 18–20 hours prior to machine perfusion.

For all groups, the Liver Assist perfusion machine (XVIVO-Abdominal, Groningen, The Netherlands) was used. This perfusion machine consists of a hepatic artery pump and a portal vein pump, both equipped with pressure-controlled centrifugal pumps and connected to hollow fiber oxygenators for dual oxygen reperfusion^46^. The machine is also primed with two liters of pre-cooled Belzer MPS UW Machine Perfusion Solution (Bridge To Life).

All livers underwent machine perfusion as follows:

- One hour of dual hypothermic machine perfusion (DHOPE) for preservation of the liver.
- One hour with controlled oxygenated rewarming (COR) to facilitate a smooth transition from hypothermia to normothermia. The livers was slowly warmed up to 37.5 $^{\circ }$C over a period of one hour. This rewarming phase links DHOPE and NMP phases, to help avoiding sudden temperature changes that might cause additional injury to the liver^41^.
- Four h﻿ours with normothermic machine perfusion (NMP).

The following perfusate solutions were applied in the various phases:

- DHOPE: Pre-cooled Bridge To Life Belzer MPS UW Machine Perfusion Solution.
- COR and NMP: The perfusate solution used during COR and NMP were based on the perfusion fluid of the Groningen DHOPE-COR-NMP protocol^10,41^. A mix of albumin, modified parenteral nutrition, multivitamins for infusion, concentrated trace elements, fast-acting insulin, calcium, sodium bicarbonate and heparin was added to a base of Leukocyte Depleted Whole Blood (LDWB) (composition of the perfusion solution is designed to provide a fluid with physiological colloid osmotic pressure and osmolality). The Groningen protocol has for some time used a hemoglobin-based oxygen carrier (Hemopure) and plasma. We used the pig’s LDWB as oxygen carrier. The component of perfusion solution used during COR and NMP is listed in details in the supplementary file.

During COR, the hepatic artery pressure started with 25 mmHg and was then increased with 10 mmHg every 15 minutes until we reached the upper arterial pressure limit set in our protocol of 55 mmHg by the end of the COR phase (i.e. start of the NMP). The portal vein pressure started at 3 mmHg in the COR phase, was increased to 5 mmHg after 30 min, to 6 mmHg at 45 min and to 7 mmHg at the start of the NMP. Both the hepatic artery pressure and the portal vein pressure were left unchanged throughout the NMP.

During DHOPE, FiO$_2$ flow was fixed at 100% and gas flow was fixed to a total of 1L/min. During both COR and NMP, FiO$_2$ and gas flow were adjusted according to the arterial partial pressure of oxygen (pO$_2$) and venous saturation at the level of the hepatic vena cava, where arterial pO$_2$ aiming range was 10.0-13.3 kPa and venous saturation 55%-75%.

Permittivity measurements were started when the liver temperature reached 37.5 $^{\circ }$C (one hour into NMP) for all groups. Measurements were performed once every hour, during the four-hour in vivo phase and the four-hour NMP phase.

### Permittivity measurements

DRS was performed with a vector network analyzer (VNA) and an open-ended coaxial probe (OCP)^47^. We used the DAK 3.5 (200 MHz–20 GHz) probe (Schmid & Partner Engineering AG, Switzerland) and the R140 (85 MHz–14 GHz) VNA (Copper Mountain Technologies). Combining the frequency limitations of the OCP and the VNA, the frequency range for our experimental setup was 200 MHz–14 GHz. A three point calibration was performed prior to each measurement session (“Open”, “Short”, and “Load” using deionized water)^48,49^. A 0.1 mol/L saline solution was used as verification liquid^19^. Calibration precision was validated by comparing the measured dielectric properties of saline to the reference published data. The maximum difference between our measured data and the reference data with respect to saline was ±2%. A new reference liquid was used for each experiment to ensure the accuracy of the calibration. Calibration was re-performed prior to each hourly measurement to maintain the accuracy of the VNA and minimize the drift error. Another systematic error associated with the performance of the system is the cable tolerance. This error is assumed to be negligible since the VNA and the probe are mechanically well fixed (without any movable cables between the VNA and the probe) during the whole experiment.

**Figure 5:**
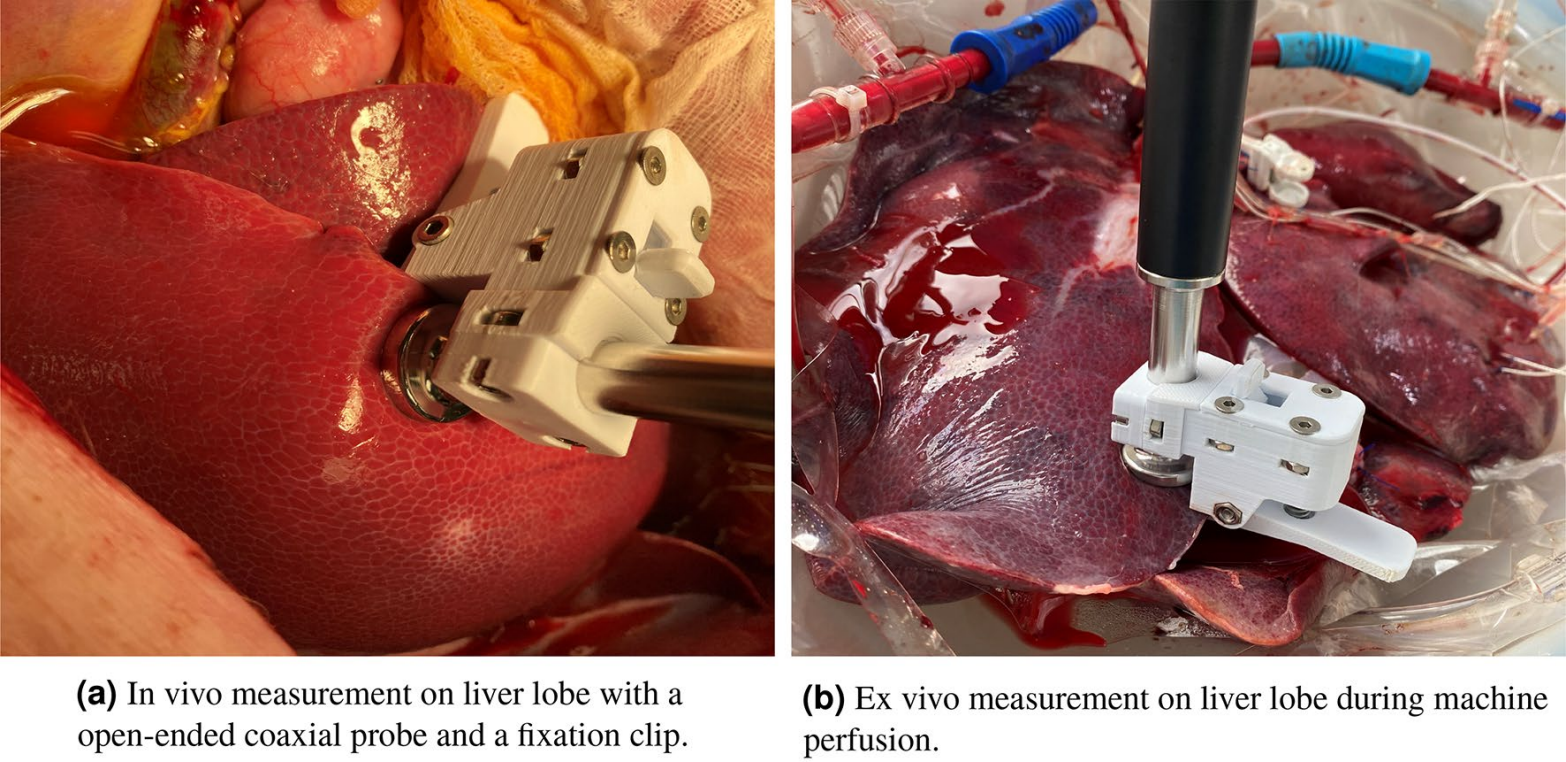
Experimental setup for both in vivo and ex vivo measurements.

To provide an accurate characterization of the dielectric parameters, 20 measurements were made, on two different random locations of the liver (10 measurements on each location), to obtain high measurement accuracy. The measured data (10 $\times$ 2) were averaged over the measured frequency range for each of the livers. To ensure that the same pressure was applied to all liver samples, a metal stand that holds both the VNA and the probe and a 3D printed clip that fits the tip of the coaxial probe were used. The liver lobe was slightly clamped between the clip and the probe surface to ensure good contact (avoid partial contact and air bobbles) and constant pressure. Erroneous measurements resulting from probe movements were efficiently minimized with the rigid support of the stand. Prior to each measurement, the measurement site was gently wiped with a sterile cloth to avoid body fluid oozing under the probe contact area and any possible contamination. After placing the probe on the liver tissue, we waited 30 seconds before starting the measurement. This was to ensure an equilibrium between the temperature of the probe tip and the liver tissue. Figure 5 shows the experimental setup for both in vivo and ex vivo measurements.

### Permittivity data analysis

Two processes (energy storage and energy dissipation) take place when the material interacts with the microwave. Dipoles in the biological sample polarize in response to the alternating electric field causing energy storage. Complex permittivity is used to express the dielectric properties of a material as Eq. (1) shows^50^.

1 $$\begin{aligned} \varepsilon ^{*} = \varepsilon ' - j\varepsilon '' = \varepsilon ' - j \frac{\sigma }{\varepsilon _{0} 2\pi f} \end{aligned}$$

where the real part $\varepsilon '$ is the dielectric constant which represents the energy storage and the imaginary part $\varepsilon ''$ is the dielectric loss which represents the energy dissipation (dissipated in the form of heat). $\sigma$ is the dielectric conductivity which represents the energy loss associated with the dispersion of $\varepsilon ''$, whenever “conductivity” is mentioned in this paper, it refers to “dielectric conductivity” which is proportional to the energy loss (Eq. (1)). $\varepsilon _0$ is the vacuum permittivity.

Relative change in the conductivity was calculated as follows:

2 $$\begin{aligned} \Delta \sigma = \frac{\sigma - \sigma _{\text {control}}}{\sigma _{\text {control}}} \times 100\%. \end{aligned}$$

The reference data ($\sigma _{\text {control}}$) used was measured in vivo, One hour after the liver was made accessible through midline laparotomy in the abdominal cavity. The reference data represents the dielectric properties of the liver in the in vivo phase, where the liver is perfused and have a stable temperature and humidity. For the rest of the data analysis, the following procedures were performed; we first averaged all measurements that had been performed at different locations on the same liver into one average representing that individual liver. We then averaged these individually representative averages from all livers within the same group, to calculate the standard error (N = 7 for each group). For statistical analysis, two-way ANOVA with multiple comparisons and Sidak correction were used.

To simplify the expression of measured permittivity data, a two-pole Cole–Cole equation^51^ (Eq. (3)) was used to model the experimental data, and thus estimate the dielectric parameter values. When water molecules are mixed with other biological substances, there is a broadening of the spectrum which corresponds to a distribution of relaxation times. Therefore, the Cole–Cole model was chosen as it takes account of the distribution of relaxation times when used to describe the dielectric relaxation of biological materials. In the liver tissue, there are protein-bound water, proteins and organic acids like lactate, which will contribute to the measured dielectric properties in addition to the contribution from water molecules^52–54^. Considering that the dielectric properties in the measurement frequency range (200 MHz–14 GHz) include both the $\beta$- and $\gamma$-dispersion, and the dispersion regions overlap^55^, we choose to use a two-pole Cole–Cole model. The chosen model takes account of both the contribution from water molecules that dominate in the higher frequency region and other molecules which are larger than water molecules that dominate in the lower frequency region. The Levenberg-Marquardt algorithm based on the non-linear least-square method was used during fitting. The two-pole Cole–Cole model can be written as:

3 $$\begin{aligned} \varepsilon ^{*}(\omega ) = \varepsilon _{\infty } + \frac{\Delta \varepsilon _{1}}{1+(j\omega \tau _1)^{1-\alpha _{1}}} + \frac{\Delta \varepsilon _{2}}{1+(j\omega \tau _2)^{1-\alpha _{2}}} \end{aligned}$$

where $\Delta \varepsilon _1 = \varepsilon _{\text {s}} - \varepsilon _1$ and $\Delta \varepsilon _2 = \varepsilon _1 - \varepsilon _\infty$ are the relaxation strength. The relaxation strength is proportional to the area under the dielectric loss peak. $\tau _1$ and $\tau _2$ are the relaxation times. $\tau$ provides information of molecular motions and their relations to the molecule sizes, shapes and the internal friction between molecules caused by the intramolecular forces. The parameters $\varepsilon _\infty$, $\tau _1$, $\tau _2$, $\Delta \varepsilon _1$, $\Delta \varepsilon _2$, $\alpha _1$, $\alpha _2$ can be obtained by fitting the experimental permittivity data to Eq. (3). The averaged permittivity data from each individual liver was used to fit to the Cole–Cole model (Eq. (3)). Seven curve fittings were performed for data from each of the injury groups and the extracted Cole–Cole parameters together with the uncertainty estimates are reported in the supplementary material.

Quantification of water content in percent was performed based on the measured dielectric loss of the deionized water, and the measured dielectric loss data of the liver. The ratio of water contribution in the liver and water contribution of the deionized water was used to estimate the water content as Eq. (4) shows:

4 $$\begin{aligned} \text {water content} [\%] = \frac{\sum [\varepsilon '' (\text {water contribution in liver})]}{\sum [\varepsilon '' (\text {deionized water})]} \times 100\% \end{aligned}$$

By summing over the dielectric loss curve for the measured deionized water ($\sum$[$\varepsilon ''$ (deionized water)]) and the separated water contribution in the liver ($\sum$[$\varepsilon ''$ (water contribution in the liver)]) from the separated high frequency dispersion using the two-pole Cole–Cole model, we were able to estimate the water content.

## Supplementary Information

Supplementary Table S1.

Supplementary Table S2.

Supplementary Figures.

## Data Availability

The datasets used and analysed during the current study available from the corresponding author on reasonable request.
