# Supplementary material for: Utilization of dielectric properties for assessment of liver ischemia-reperfusion injury in vivo and during machine perfusion
Source: Sci Rep. 2022 Jul 1;12:11183. doi: 10.1038/s41598-022-14817-3 (PMC9249774; doi:10.1038/s41598-022-14817-3)
Supplement: Supplementary file 3 — Supplementary Figures. [file 41598_2022_14817_MOESM3_ESM.pdf]

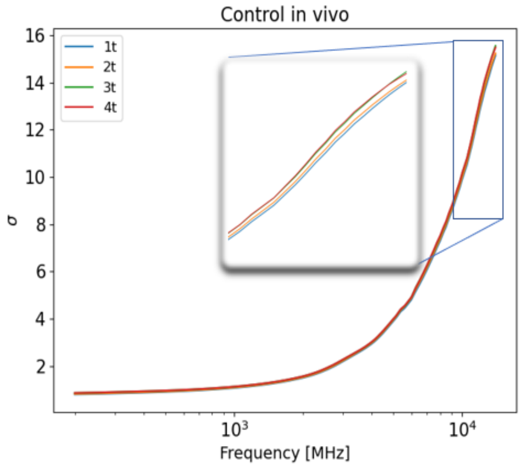

(a) Control group during in vivo monitoring.

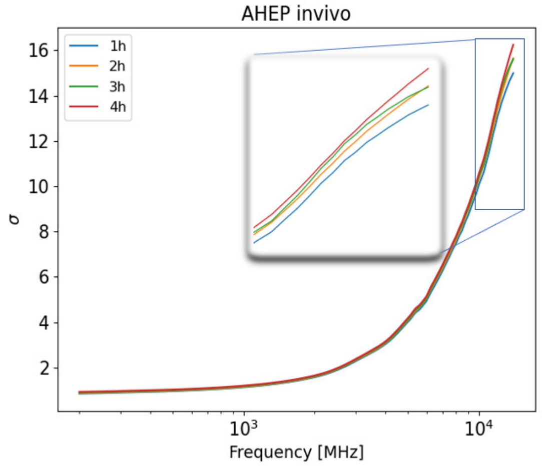

(b) AHEP group during in vivo monitoring.

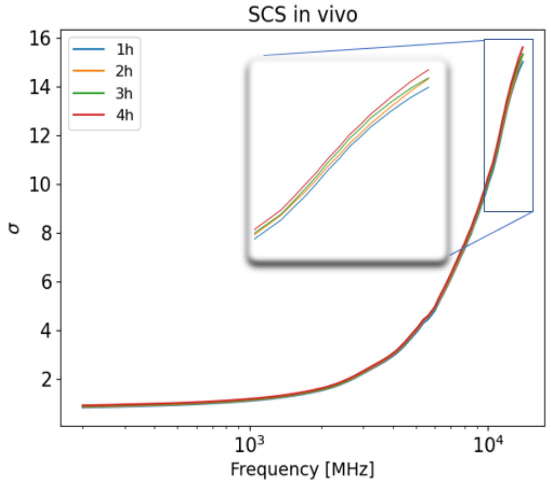

(c) SCS group during in vivo monitoring.

Figure S1. Raw conductivity ($\sigma[S/m]$) data for the three groups (N = 7 for each of the groups) as a function of frequency (200 MHz - 14 GHz) during in vivo phase.

The measured raw conductivity data both in vivo and on NMP for the control, AHEP and SCS groups, respectively, are shown in Figure S1 and S2. There are noticeable differences in the high frequency region as a function of time.

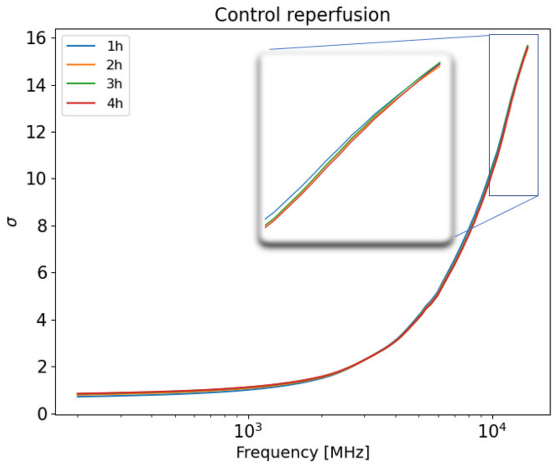

(a) Control group during NMP.

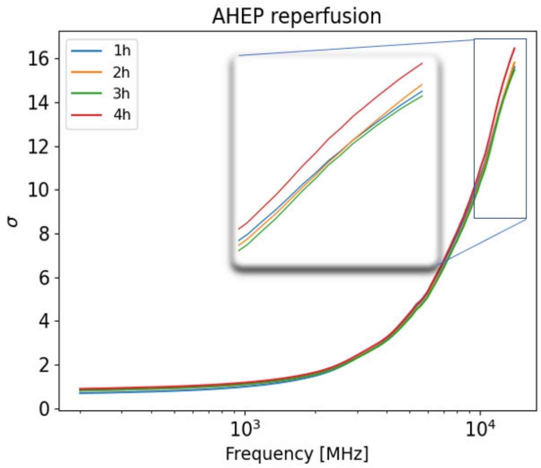

(b) AHEP group during NMP.

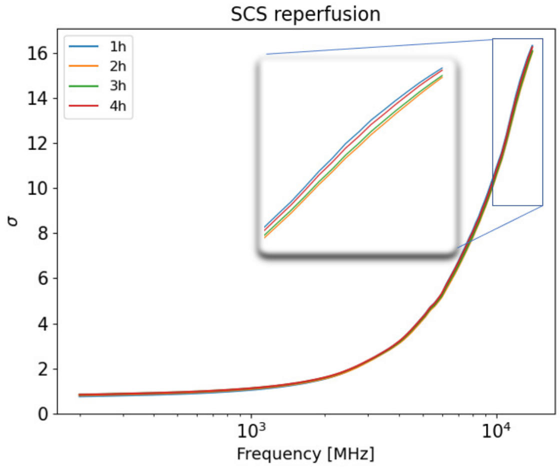

(c) SCS group during NMP.

Figure S2. Raw conductivity ($\sigma[S/m]$) data for the three groups (N = 7 for each of the groups) as a function of frequency (200 MHz - 14 GHz) during four hours monitoring on NMP.
